# Comparison of platelet-and endothelial-associated biomarkers of disease activity in people hospitalized with Covid-19 with and without HIV co-infection

**DOI:** 10.3389/fimmu.2023.1235914

**Published:** 2023-08-14

**Authors:** Mieke A. van der Mescht, Helen C. Steel, Zelda de Beer, Fareed Abdullah, Veronica Ueckermann, Ronald Anderson, Theresa M. Rossouw

**Affiliations:** ^1^ Department of Immunology, Faculty of Health Sciences, University of Pretoria, Pretoria, South Africa; ^2^ Department of Family Medicine, Tshwane District Hospital, Pretoria, South Africa; ^3^ Division for Infectious Diseases, Department of Internal Medicine, Steve Biko Academic Hospital and University of Pretoria, Pretoria, South Africa; ^4^ Office of AIDS and TB Research, South African Medical Research Council, Pretoria, South Africa

**Keywords:** SARS-CoV-2, COVID-19, HIV, platelets, cytokines, chemokines, vascular endothelial growth factor

## Abstract

**Introduction:**

SARS-CoV-2 elicits a hyper-inflammatory response that contributes to increased morbidity and mortality in patients with COVID-19. In the case of HIV infection, despite effective anti-retroviral therapy, people living with HIV (PLWH) experience chronic systemic immune activation, which renders them particularly vulnerable to the life-threatening pulmonary, cardiovascular and other complications of SARS-CoV-2 co-infection. The focus of the study was a comparison of the concentrations of systemic indicators o\f innate immune dysfunction in SARS-CoV-2-PCR-positive patients (n=174) admitted with COVID-19, 37 of whom were co-infected with HIV.

**Methods:**

Participants were recruited from May 2020 to November 2021. Biomarkers included platelet-associated cytokines, chemokines, and growth factors (IL-1β, IL-6, IL-8, MIP-1α, RANTES, PDGF-BB, TGF-β1 and TNF-α) and endothelial associated markers (IL-1β, IL-1Ra, ICAM-1 and VEGF).

**Results:**

PLWH were significantly younger (p=0.002) and more likely to be female (p=0.001); median CD4+ T-cell count was 256 (IQR 115 -388) cells/μL and the median HIV viral load (VL) was 20 (IQR 20 -12,980) copies/mL. Fractional inspired oxygen (FiO2) was high in both groups, but higher in patients without HIV infection (p=0.0165), reflecting a greater need for oxygen supplementation. With the exception of PDGF-BB, the levels of all the biomarkers of innate immune activation were increased in SARS-CoV-2/HIV-co-infected and SARS-CoV-2/HIV-uninfected sub-groups relative to those of a control group of healthy participants. The magnitudes of the increases in the levels of these biomarkers were comparable between the SARS-CoV-2 -infected sub-groups, the one exception being RANTES, which was significantly higher in the sub-group without HIV. After adjusting for age, sex, and diabetes in the multivariable model, only the association between HIV status and VEGF was statistically significant (p=0.034). VEGF was significantly higher in PLWH with a CD4+ T-cell count >200 cells/μL (p=0.040) and those with a suppressed VL (p=0.0077).

**Discussion:**

These findings suggest that HIV co-infection is not associated with increased intensity of the systemic innate inflammatory response during SARS-CoV-2 co-infection, which may underpin the equivalent durations of hospital stay, outcome and mortality rates in the SARS-CoV-2/HIV-infected and -uninfected sub-groups investigated in the current study. The apparent association of increased levels of plasma VEGF with SARS-CoV-2/HIV co-infection does, however, merit further investigation.

## 1 Introduction

Cytokine release syndrome, also referred to as hypercytokinemia or a ‘cytokine storm’, is a consequence of a dysregulated immune response and has been linked to the pathology seen in individuals presenting with severe COVID-19 (1). High levels of pro-inflammatory cytokines cause symptoms such as fever, hemodynamic instability, coagulopathy, splenomegaly, hepatitis, and multi-organ failure, which can be fatal (1). One of the common causes of mortality in COVID-19 is hypercoagulability (2), with some patients presenting with strokes, myocardial infarcts, pulmonary emboli, mesenteric ischemia, and limb thrombosis (3). Microemboli have also been associated with myocarditis and cardiac failure (2).

While it is still unclear whether the virus itself has intrinsic procoagulant effects, coagulopathy likely stems from the inflammatory response observed in these individuals and the ensuing endothelial activation or damage (4). Some authors have suggested that COVID-19 should be viewed and treated as a ‘true vascular disease’ and the importance of endothelium-platelet interactions is increasingly recognised (5, 6).

Although the main function of platelets is to maintain vascular integrity through coagulation and angiogenesis, their role in both the innate and adaptive immune response is also well recognised (7). Platelets are involved in the first-line response to invading pathogens, activating an innate immune response through Toll-like receptors (TLRs) (7). Toll-like receptor-7 plays an important role in protecting against viral infections in the innate immune system (8). SARS-CoV-2, a single-stranded ribonucleic acid (ssRNA) virus, binds to TLR-7 within endosomal compartments of cells, including platelets (9). Following binding of ssRNA viruses to TLR-7, the MyD88-dependent pathway is initiated, resulting in upregulated expression of inflammatory cytokines, most notably interleukin (IL)-1, IL-6, IL-10, IL-12, and tumor necrosis factor (TNF)-α, by various immune cells including T-cells, B-cells, macrophages, monocytes, fibroblasts, plasmacytoid dendritic cells, and endothelial cells (10). The upregulated expression of these cytokines is consistent with a skewing of the immune response towards a T helper (Th)2 or Th17 phenotype (10).

It has been shown that platelets interact directly with SARS-CoV-2 and enhance uptake of the virus into various cell types (9). Platelets are hyperactivated during SARS-CoV-2 infection through mechanisms as diverse as epithelial damage, hypoxia, neutrophil extracellular trap formation, interactions between SARS-CoV-2 spike protein and platelets, autoimmune responses, and autocrine activation (6). Activated platelets undergo degranulation and release a number of inflammatory mediators that are stored in their granules (11). In addition, activated platelets also exhibit upregulated expression of adhesion molecules, including platelet-derived growth factor (PDGF) and regulated upon activation normal T-cell expressed and secreted (RANTES), which are both stored in and secreted from platelet α-granules (12).

South Africa has one of highest prevalences of HIV infection in the world, estimated at 13% in the general and 18.7% in the adult population (13). From the start of the pandemic, it was feared that HIV-associated immunodeficiency might predispose people living with HIV (PLWH) to infection with SARS-CoV-2, and that the persistent, systemic immune activation caused by HIV might exacerbate hyperinflammation during COVID-19. Both scenarios could lead to poor outcomes. Notably, an increase in thrombotic events (up to ten-fold higher) has been reported in PLWH receiving antiretroviral therapy (ART) compared to those of the healthy, uninfected population (14). This vulnerable population is, therefore, potentially at an even higher risk of developing thrombosis if co-infected with SARS-CoV-2.

Nevertheless, despite the existence of an extensive body of literature, predominantly encompassing clinical studies and systematic reviews focused on comparisons of outcomes of patients hospitalized with COVID-19 without and with HIV co-infection, few of these studies have included detailed comparisons of the levels of biomarkers of systemic innate immune activation and their associations with organ dysfunction and outcome. To address this issue, the present study compared associations between the systemic levels of biomarkers of platelet and endothelial activation with clinical parameters and treatment outcomes in people living with and without HIV infection, admitted to hospital with COVID-19 in Tshwane, South Africa.

## 2 Materials and methods

### 2.1 Study population and sample collection

In this study, 174 patients who were admitted with COVID-19 (parameters of disease severity can be found in **Supplementary Table 1**) were recruited from May 2020 until December 2021 from Steve Biko Academic and Tshwane District Hospitals, Pretoria, South Africa. For each patient, approximately 20 milliliters (mL) of blood were collected in tubes containing ethylenediaminetetraacetic acid (EDTA) as anticoagulant on the first day of admission, before treatment was commenced. The inclusion criteria were as follows: SARS-CoV-2 polymerase chain reaction (PCR)-positive; 18 years or older; and willing and able to provide informed consent to participate. Samples were processed and stored on the day of venipuncture. Plasma was stored in sterile tubes at -80°C until assayed. Results of routine pathology tests were extracted from the National Health Laboratory Service (NHLS) Trakcare database. Clinical data were recorded by trained clinicians using RedCap (v9.5.36). The study was approved by the University of Pretoria Faculty of Health Sciences Research Ethics Committee (ref. 247/2020). Nine healthy volunteers with no prior history of SARS-CoV-2 or HIV were included as controls for biomarker testing.

### 2.2 Quantification of cytokines, chemokines, and growth factors

Circulating levels of cytokines were determined in plasma samples using a Bio-Plex Human Cytokine/Chemokine Magnetic Bead Panel Kit (Bio-Rad Laboratories, Inc., Hercules, CA, USA). The concentrations of the following cytokines, chemokines, and growth factors were determined: interleukin (IL)-1β, IL1-receptor antagonist (IL-1Ra), vascular endothelial growth factor A (VEGF-A, also known as just VEGF), IL-6, tumor necrosis factor (TNF)-α, macrophage inflammatory protein-1 alpha (MIP-1α), platelet-derived growth factor (PDGF)-BB, and IL-8. Plasma samples were diluted four-fold and the experimental procedure was followed as per the manufacturer’s instructions. Briefly, prepared magnetic beads (50 µL) were added to each of the 96 wells, and the plate was washed twice using an automated magnetic microplate washer (Bio-Rad Laboratories, Inc.). The diluted samples, blanks, standards, and controls (50 µL) were added to appropriately designated wells. The plate was sealed and incubated (protected from light) at room temperature for 45 minutes with gentle agitation on an orbital plate shaker (Thomas Scientific, Swedesboro, NJ, USA). Following the incubation period, the plate was washed three times as described above and detection antibody (25 µL) was added to each well. The plate was sealed and incubated for 30 minutes at room temperature with gentle agitation. The plate was again washed three times followed by the addition of streptavidin-phycoerythrin (50 µL). The plate was sealed and incubated for 15 minutes at room temperature with gentle agitation. The plate was then washed a final three times. The beads were resuspended in 125 µL assay buffer and shaken vigorously for two minutes on a Cooke AM69 microplate shaker (Dynatech AG, Bleichestrasse, ZUG, CH) prior to assay on a Bio-Plex Suspension Array platform (Bio-Rad Laboratories, Inc.). Bio-Plex Manager Software 6.0 was used for bead acquisition and analysis of median fluorescence intensity. Results are presented as picograms per milliliter (pg/mL).

#### 2.2.1 Preparation and dilution of: regulated-on activation, normal T-cell expressed and secreted, intracellular-adhesion molecule-1, and transforming growth factor-β1

The regulated-on activation, normal T-cell expressed and secreted (RANTES) concentrations were determined using the Human RANTES enzyme-linked immunoassay (ELISA) kit (E-EL-H6006, Elabscience Biotechnology, Inc., Houston, TX, USA). Intercellular adhesion molecule 1 (I-CAM-1) levels were measured using the Human I-CAM-1/CD54 ELISA kit (E-EL-H6114, Elabscience, Biotechnology, Inc.). Samples for both assays were thawed at room temperature and diluted to a ratio of 1:20 prior to analysis.

Transforming growth factor-β1 (TGF-β1) levels were determined using the Human TGF-β1 ELISA kit (E-EL-0162, Elabscience Biotechnology, Inc.). Latent TGF-β1 was activated to the immunoreactive form by adding 40 µL 1N hydrochloric acid to 240 µL plasma (diluted 1:8). After mixing thoroughly, the samples were incubated for 10 minutes at room temperature. The samples were then neutralized with the addition of 40 µL 1.2 N sodium hydroxide and mixing thoroughly. The assay was performed immediately.

#### 2.2.2 Procedure for sandwich enzyme-linked immunosorbent assays

Levels of RANTES, I-CAM-1 and TGF-β1 were determined as per the manufacturer’s instructions described briefly below.

The standards and appropriately prepared plasma samples (100 µL) were added to the designated wells. The plate was sealed and incubated for 1.5 hours at 37°C. Following the incubation period, the plate contents were discarded followed by the addition of biotinylated detection antibody (100 µL) to each well and the plate incubated for 1 hour at 37°C. The plate was then washed three times using an automated plate washer (BioTek Instruments, Inc., Winooski, VT, USA) followed by the addition of horseradish peroxidase (HRP) conjugate (100 µL) to each well. The plate was incubated for an additional 30 minutes at 37°C. The plate was washed a further five times as described above followed by the addition of 3,3’,5,5’-tetramethylbenzidine (TMB) substrate reagent (100 µL) and a further 15-minute incubation period at 37°C, protected from light. The reaction was stopped by the addition of 50 μL stop solution and the optical density (OD) read at a wavelength of 450 nm using a plate spectrophotometer (BioTek Instruments Inc.). The concentration of the analyte present in each sample was determined using the appropriate generated standard curve and the results are presented as nanograms (ng)/mL.

### 2.3 Statistical analysis

Clinical information was captured from patient files and entered into a Microsoft excel spreadsheet. Results of routine laboratory tests were obtained from the NHLS. Results from specialized immunological tests were exported from the instrument to the spreadsheet. Double data entry by two independent researchers ensured the accuracy of the records. Data were exported to Stata 17 for analysis. Data were assessed for distribution and appropriate tests applied. The student’s t-test and Kruskal-Wallis test were used to compare continuous variables between groups, while Pearson’s chi-square and Fisher’s exact test were used for univariate comparison of categorical variables. Stepwise, backward, multivariable logistic regression analysis was used to examine associations with HIV status after appropriate transformation of predictor variables. Spearman’s correlation test, with Bonferroni correction for multiple comparisons, was used to assess correlations between continuous variables.

## 3 Results

### 3.1 Demographic and clinical parameters at the time of hospital admission

A total of 174 patients was recruited. Their mean age was 52 (SD ±14) years and just over half (53.5%) were male. Comorbidities were common: hypertension (42.8%), diabetes mellitus (34%), heart disease (14.5%), kidney disease (9.9%), lung disease (8.9%), and cancer (3.1%). Of the group, 37 (21%) were PLWH. Twenty-six (70.3%) PLWH were on ART, with one having started 3 days before admission. The median CD4+ T-cell count was 256 (IQR 115 – 388) cells/μL and the median HIV viral load (VL) was 20 (IQR 20 – 12,980) copies/mL. Nineteen PLWH (51.4%) had a suppressed VL (i.e. VL <20 copies/mL) at the time of admission. PLWH were significantly younger and more likely to be female. They were also less likely to have diabetes mellitus as a comorbidity, but more likely to have had, or to currently have, active TB. There was no difference in their duration of stay in the hospital, disease severity, or in their outcome. The in-hospital mortality rate for the cohort was 18.6%. The mortality rate in the PLWH group was 12.5% compared to 20% in the non-PLHIV group and the difference was not significant. These data are summarized in **Table 1**.

**Table 1:** Clinical characteristics of the participants.

Variable		COVID-19 PLWH (n=37)	COVID-19 HIV-uninfected (n=137)	P-value
Age (years)		45.6 ( ± 11)	53.7 ( ± 14.6)	**0.002**
Gender (male)		11/37 (29.7%)	82/137 (59.9%)	**0.001**
Hypertension		12/37 (32.4%)	56/122 (45.9%)	0.147
Diabetes		6/37 (16.2%)	48/122 (45.9%)	**0.009**
Heart disease		4/37 (10.8%)	19/122 (15.6%)	0.599
Lung disease		2/37 (5.4%)	12/121 (9.9%)	0.523
Kidney disease		2/37 (5.4%)	15/125 (12%)	0.080
Cancer		2/37 (5.4%)	3/122 (2.5%)	0.330
Current TB		3/36 (8.3%)	1/125 (0.8%)	**0.015**
Past TB		5/36 (13.8%)	2/125 (1.6%)	**0.004**
Overweight		9/35 (25.7%)	36/117 (30.8%)	0.256
Duration of admission		8 (5 – 10)	8 (5 – 12)	0.722
Disease severity:	mild moderate severe	13/35 (37.1%) 18/35 (51.4%) 4/35 (11.4%)	23/114 (20.2%) 62/114 (54.4%) 29/114 (25.4%)	0.061
Outcome (died)		4/32 (12.5%)	22/108 (20%)	0.395

Age is shown as mean (± standard deviation) and duration of admission as median (interquartile range). All other variables are shown as number (percentage).

People living with HIV (PLWH), Tuberculosis (TB).

Values in bold are significant.

With respect to cardiac and pulmonary function on admission, both groups had an elevated pulse and respiratory rate. Patients without HIV had lower levels of saturation on room air, although this association just missed statistical significance. Fractional inspired oxygen (FiO2) was high in both groups, but significantly higher in patients without HIV infection, reflecting a greater need for oxygen supplementation. The ratio of arterial oxygen partial pressure (PaO2 in mmHg) to FiO2, known as the PF ratio, was low in both groups, indicating moderate acute respiratory distress syndrome (ARDS) (**Supplementary Table 2**).

Access to specialized scans was unfortunately limited during the pandemic and only ten cases of clinically detectable thrombotic events were reported: eight in people without HIV (8/137 - 5.8%) and two in PLWH (2/37 - 5.4%) (p=0.640).

Routine blood parameters measured at the time of hospital admission are shown in **Table 2**. PLWH and HIV-uninfected patients had significantly elevated, but equivalent, levels of C-reactive protein (CRP). While ferritin levels were elevated in both groups, PLWH had significantly lower levels of this biomarker. In addition, D-dimers were similarly elevated in both groups, with no differences observed between the groups. Apart from significantly lower hemoglobin levels in PLWH, all other hematological markers were similar. All patients had a notable lymphopenia, but platelet counts were within normal limits. A total of 16 patients had thrombocytopenia: 4 living with and 12 without HIV (p=0.714).

**Table 2:** Routine blood parameters at admission.

Variable	COVID-19 PLWH (n=37)	COVID-19 HIV-uninfected (n=137)	Reference range	P-value
CRP (mg/L)	116 (57 – 189.5	105.5 (53 – 195)	<10	0.7819
Ferritin (ug/L)	269.5 (79 – 855)	742.5 (325 – 1576)	5 – 204	**0.0020**
D-dimer (mg/L)	0.77 (0.5 – 5)	0.66 (0.4 – 1.5)	0.00 – 0.25	0.2523
Trop I (ng/L)	10 (10 – 24)	11 (10 - 35)		0.4390
Hb (g/dL)	11.4 (± 2.96)	13.5 (± 2.6)	11.6 – 16.4	**0.0001**
Platelets (L x 10^9^/L)	277.5 (208– 359)	263 (202.5 – 324.5)	186 – 454	0.4841
WCC (H x 10^9^/L)	7.8 (5.7 – 9.8)	9.3 (6.8 – 11.8)	3.90 – 12.60	0.0509
Neutrophil count (H x 10^9^/L)	6.8 (5.4 – 7.7)	6.8 (4.7 – 10.3)	1.60 – 8.30	0.4214
Lymphocyte count (L x 10^9^/L)	1.06 (0.75 – 1.61)	1.16 (0.74 – 1.75)	1.40 – 4.50	0.9271
NLR	6.4 (2.8 – 9.7)	6.4 (3.5 – 10.3)		0.7959
PNR	43.2 (32.2 – 70.5)	38.9 (17.0 – 37.1)		0.2970
PLR	310.8 (156.7 – 419.3)	253.9 (153.6 – 347.1)		0.3921

All variables are shown as median (interquartile range) except for Hb which is shown as mean (± standard deviation)

C-reactive protein (CRP), Hemoglobin (Hb), neutrophil lymphocyte ratio (NLR), platelet lymphocyte ratio (PLR), People living with HIV (PLWH), platelet neutrophil ratio (PNR), troponin I (Trop I), white cell count (WCC).

Values in bold are significant.

### 3.2 Systemic concentrations of biomarkers of platelet and endothelial activation

Cytokines, chemokines, and growth factors representative of platelet (IL-8, MIP-1α, RANTES, IL-1β, IL-6, TNF-α, PDGF-BB, TGF-β1) and endothelial cell (VEGF, IL-1Ra, IL-1β, ICAM-1) activation were compared between patients with COVID-19, with and without HIV infection, and healthy control participants (n=9) by means of univariate analysis. The median age of the control group was 44 years (IQR 41 – 47) with a male:female ratio of 2:1. As shown in **Table 3**, all biomarkers, with the exception of PDGF-BB, were significantly different between the three groups with all markers being higher in patients with COVID-19, with or without HIV infection, than in controls. The only significant difference observed between patients with COVID-19 with and without HIV infection was RANTES, which was significantly lower in PLWH (p=0.034). RANTES levels were significantly higher in people with acute kidney disease [117.78 (36.42-166.66) p=0.0435] and narrowly missed significance in patients with hypertension [96.41 (46.17-163.92), p=0.0511]. In patients with cancer, RANTES levels were significantly lower [56.76 (23.32-64.99), p=0.040]. Male patients had significantly higher levels of RANTES [118.53 (54.17-204.44)] than female patients [68.19 (42.11-114.51), p=0.0016]. The results of the linear regression indicated that age explained 3.35% of the variation in RANTES levels [F(1,169)= 6.89, p=0.0095].

**Table 3:** Immunological parameters at admission.

Variable	COVID-19 PLWH (n=37)	COVID-19 HIV-uninfected (n=137)	Controls (n=9)	P-value*
ICAM-1 (ng/mL)	153.88 (132.09 – 170.56)	144.19 (121.61 – 183.81)	112.23 (78.97 – 112.36)	**0.0099**
TGF-β1 (ng/mL)	7.35 (5.2 – 11.57)	8.08 (5.82 – 11.85)	5.02 (3.10 – 5.73)	**0.0476**
RANTES (ng/mL)	65.10 (51.62 – 104.93)	98.93 (45.63 – 181.51)	57.29 (36.51 – 60.35)	**0.0371**
IL-1β (pg/mL)	1.95 (1.46 – 2.66)	1.95 (1.46 – 2.89)	1.22 (0.98 – 1.71)	**0.0492**
IL-1Ra (pg/mL)	894.53 (725.59 – 1133.51)	812.8 (631.27 – 1089.68)	237.73 (214.08 – 407.8)	**0.0002**
IL-6 (pg/mL)	5.79 (1.82 – 12.0)	4.32 (1.6 – 9.4)	0.49 (0.3 – 0.54)	**0.0007**
IL-8 (pg/mL)	19.69 (15.32 – 28.36)	17.5 (10.73 – 23.48)	3.58 (2.8 – 6.47)	**0.0001**
MIP-1α (pg/mL)	4.10 (2.69 – 5.17)	3.29 (2.47 – 4.72)	1.3 (0.95 – 1.3)	**0.0001**
PDGF-BB (pg/mL)	460.42 (192.38 – 955.7)	504.48 (253.69 – 1044.48)	392.41 (31.74 – 494.58)	0.0926
TNF-α (pg/mL)	93.16 (73.41 – 118.40)	83.87 (68.6 – 102.39)	55.49 (50.67 – 68.6)	**0.0009**
VEGF (pg/mL)	17.71 (4.22 – 165.71)	4.22 (4.22 – 87.07)	4.22 (4.22 – 4.22)	**0.0480**

All variables are shown as median (interquartile range).

intracellular-adhesion molecule-1 (ICAM-1), transforming growth factor-β1 (TGF-β1), regulated-on activation, normal T-cell expressed and secreted (RANTES), interleukin (IL), IL1-receptor antagonist (IL-1Ra), macrophage inflammatory protein-1 alpha (MIP-1α), platelet-derived growth factor BB (PDGF-BB), tumor necrosis factor-α (TNF-α), vascular endothelial growth factor (VEGF).

*P-values represent the overall difference between the 3 groups according to the Kruskal-Wallis test. Values in bold indicate significance. P-values for the differences between respective groups, according to the post-hoc Dunn test, are shown in **Supplementary Table 1**.

Stepwise backward multivariable logistic regression of all the markers revealed significant associations between HIV and PDGF-BB (OR=0.65; p=0.035), as well as between HIV and VEGF (OR=1.32; p=0.034). After adjusting for age, sex, disease severity, and diabetes in the multivariable model, the only significant association that remained was between HIV status and VEGF (**Table 4**). Levels of VEGF were higher in people living with hypertension (28.75 [IQR 4.22 - 150.01] versus 4.22 [IQR 4.22 - 72.88]; p=0.0312) but not in those with diabetes (p=0.2181), pre-existing lung disease (p=0.5558), kidney disease (p=0.6602) or cancer (p=0.3564).

**Table 4:** Multivariable logistic regression model.

HIV	Odds ratio	Standard Error	Z	P>z	95% confidence interval	
Age	0.9612	0.0159	-2.39	**0.017**	0.9305	0.9929
Sex	0.2580	0.1138	-3.07	**0.002**	0.1087	0.6125
Diabetes	0.2777	0.1484	-2.40	**0.016**	0.0975	0.7913
PDGF-BB	0.7800	0.1828	-1.06	0.289	0.4927	1.2347
VEGF	1.3537	0.1931	2.12	**0.034**	1.0236	1.7902
Estimated baseline odds	10.1942	14.4829	1.63	0.102	0.6296	165.0657

platelet-derived growth factor (PDGF), vascular endothelial growth factor (VEGF).

Values in bold indicate significance.

In order to determine whether the association with VEGF was due to HIV alone or rather secondary to a SARS-CoV-2/HIV interaction, PLWH with COVID-19 were compared to nine PLWH without COVID-19. The median age of this group was 47 years (IQR 39 - 61); 5 were female and 4 were male. Eight were on ART. The median CD4 count was 196 cells/mm3 (IQR 126 - 349), VL was 79 copies/mL (IQR 20 - 19,900); three had a suppressed VL (≤20 copies/mL) and six an unsuppressed VL. VEGF was significantly higher in PLWH with COVID-19 than in PLWH without COVID-19 (17.71 [IQR 4.22 - 165.71] versus 4.22 [IQR 4.22 - 4.22]; p=0.019). There was no difference in VEGF between the virally suppressed and unsuppressed PLWH without COVID-19 (p=0.2642), although it should be kept in mind that the numbers in these sub-groups were small.

**Figure 1:**
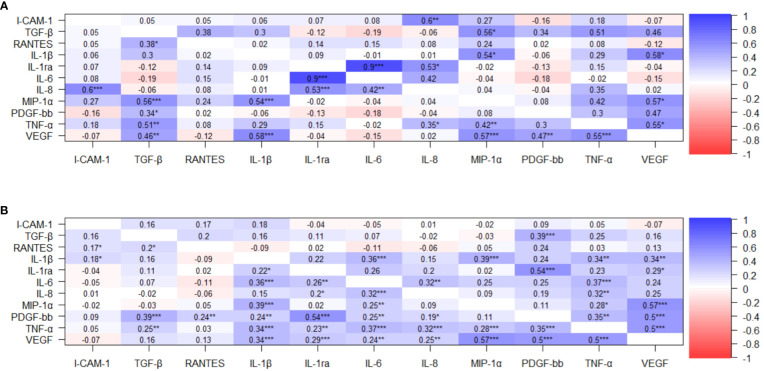
Correlation plots of cytokine interactions. **(A)** COVID-19 PLWH, statistically significant positive correlations with rho above 0.5 were identified between the following cytokines: IL-8 and ICAM-1; TGF-β1 and MIP-1α; TGF-β1 and TNF-α; MIP-1α and IL-1β; IL-1β and VEGF; IL-1Ra and IL-6; IL-1Ra and IL-8; MIP1-α and VEGF; and TNF-α and VEGF. **(B)** COVID-19 HIV-uninfected, statistically significant positive correlations with rho above 0.5 were identified between the following cytokines: IL-1Ra and PDGF-BB; MIP-1α and VEGF; PDGF-BB and VEGF; and TNF-α and VEGF. *: p < 0.05, **: p < 0.01, ***: p< 0.001.

Many variables were significantly correlated with one another, but no correlations were found between the cytokines and either the CD4+ T-cell count or the HIV VL, or between CD4 T-cell count and HIV VL. (**Supplementary Figure 1**). The heatmap presented in **Figure 1A** represents the correlations in the COVID-19/PLWH group while the heatmap in **Figure 1B** depicts the results in people with COVID-19 but without HIV.

Just under half of PLWH (17/36 – 47.2%) with CD4+ T-cell count data (36/37 – 97.3%) had a level ≤200 cells/μL. These individuals had non-significantly lower levels of saturation on O2 (94% [IQR 90.5% - 96%] versus 96% [IQR 95% - 98%]; p=0.0564), and equivalent levels of FiO2 (p=0.1647), CRP (p=0.7185) and ferritin (p=0.750) when compared with individuals with counts above 200 cells/μL. They also had significantly higher levels of IL-6 and lower levels of VEGF. Although levels of RANTES and IL-8 were both higher in PLWH with a CD4 ≤200 cells/μL, these differences missed statistical significance (**Table 5**). Four out of the 17 (23.5%) COVID-19/PLWH patients with a CD4 count ≤200 cells/μL died, compared to none of the 15 (with outcome data) and a CD4 count >200 cells/μL. Levels of all the markers tested differed significantly between COVID-19/PLWH and PLWH controls, suggesting that all the changes observed were induced by COVID-19 in the context of HIV infection.

**Table 5:** Comparison of immunological markers in PLWH with COVID-19 with low and higher CD4 counts.

Variable	COVID-19 PLWH CD4 ≤200 cells/μL (n=17)	COVID-19 PLWH CD4 >200 cells/μL (n=19)	P-value*
ICAM-1 (ng/mL)	169.192 (144.02–178.61)	153.88 (132.09 –169.78)	0.1303
TGF-β1 (ng/mL)	7.35 (5.08 –11.57)	7.98 (5.20 –17.06)	0.2736
RANTES (ng/mL)	70.22 (59.71–107.02)	64.99 (32.98–82.01)	0.0890
IL-1β (pg/mL)	2.03 (1.71–2.43)	1.95 (1.35–3.69)	0.9494
IL-1Ra (pg/mL)	952.91 (747.98–1175.93)	894.53 (655.64–1133.51)	0.6345
IL-6 (pg/mL)	13.18 (5.39–72.3)	4.32 (1.25–7.28)	**0.0028**
IL-8 (pg/mL)	23.81 (15.32–31.61)	18.59 (14.45–20.24)	0.0769
MIP-1α (pg/mL)	4.8 (3.83–5.17)	3.38 (2.69–5.57)	0.2163
PDGF-BB (pg/mL)	578.07 (222.09–920.74)	378.77 (181.27–1040.34)	0.9873
TNF-α (pg/mL)	93.16 (76.85–116.12)	84.6 (73.41–124.78)	0.8121
VEGF (pg/mL)	4.22 (4.22–72.88)	123.35 (4.22–269.27)	**0.0141**

All variables are shown as median (interquartile range).

intracellular-adhesion molecule-1 (ICAM-1), transforming growth factor-β1 (TGF-β1), regulated-on activation, normal T-cell expressed and secreted (RANTES), interleukin (IL), IL1-receptor antagonist (IL-1Ra), macrophage inflammatory protein-1 alpha (MIP-1α), platelet-derived growth factor BB (PDGF-BB), tumor necrosis factor-α (TNF-α), vascular endothelial growth factor (VEGF).

Values in bold indicate significance.

Eighteen (18/37 – 48.65%) PLWH had a detectable VL (>20 copies/mL). Compared to PLWH with an undetectable VL, they were significantly younger (39.9 ± 11 years versus 51.1 ± 7.7 years; p=0.037), less likely to have hypertension (16.7% versus 47%; p=0.046), diabetes (0% versus 31.6%, p=0.009) or be overweight (0% versus 47.4%; p=0.001). Patients with a detectable VL were also more likely to have active TB, but this difference was not statistically significant (17.6% versus 0%; p=0.095). They had significantly lower levels of TGF-β1, IL-1β, PDGF-BB, TNF-α and VEGF (**Table 6**). After multivariable logistic regression, only the association between VL and age (p=0.024) and VEGF (p=0.043) remained.

**Table 6:** Comparison of immunological markers in PLWH with COVID-19 with detectable and undetectable HIV viral loads.

Variable	COVID-19 PLWH VL >20 copies/mL (n=18)	COVID-19 PLWH VL ≤20 copies/mL (n=19)	P-value*
ICAM-1 (ng/mL)	155.59 (132.09–175.55)	162.49 (134.75–170.56)	0.4158
TGF-β1 (ng/mL)	6.49 (4.41–10.51)	9.15 (6.86–15.12)	**0.0268**
RANTES (ng/mL)	66.31 (58.65–104.93)	64.78 (26.58–83.23)	0.1370
IL-1β (pg/mL)	1.90 (1.46–2.03)	2.43 (1.47–4.93)	**0.0442**
IL-1Ra (pg/mL)	886.04 (439.52–990.82)	979.5 (844.02–1462.14)	0.0833
IL-6 (pg/mL)	4.03 (1.25–31.69)	7.99 (4.32–13.18)	0.2626
IL-8 (pg/mL)	18.29 (12.89–28.23)	20.24 (17.47–29.62)	0.2016
MIP-1α (pg/mL)	3.52 (2.02–4.87)	4.57 (2.99–5.57)	0.0605
PDGF-BB (pg/mL)	307.17 (148.02–578.07)	704.67 (343.84–1052.76)	**0.0167**
TNF-α (pg/mL)	73.96 (60.28–107.82)	113.84 (81.64–148.85)	**0.0025**
VEGF (pg/mL)	4.22 (4.22–66.99)	126.12 (4.22–269.27)	**0.0021**

All variables are shown as median (interquartile range).

intracellular-adhesion molecule-1 (ICAM-1), transforming growth factor-β1 (TGF-β1), regulated-on activation, normal T-cell expressed and secreted (RANTES), interleukin (IL), IL1-receptor antagonist (IL-1Ra), macrophage inflammatory protein-1 alpha (MIP-1α), platelet-derived growth factor BB (PDGF-BB), tumor necrosis factor-α (TNF-α), vascular endothelial growth factor (VEGF).

Values in bold indicate significance.

No significant differences in the levels of VEGF could be found between healthy PLWH controls and PLWH co-infected with SARS-CoV-2 with detectable VL. PLWH (19/37-51.35%) with an undetectable VL had significantly higher levels of VEGF than healthy PLWH controls with undetectable VL (126.12 [IQR 4.22– 269.27] versus 4.22 [IQR 4.22– 4.22]; p=0.0117) and detectable VL (126.12 [IQR 4.22– 269.27] versus 4.22 [IQR 4.22– 4.22]; p=0.0200).

All four of the PLWH who died had a CD4+ T-cell count of <200 cells/μL, 3/4 had a detectable VL, and two were known not to be on ART.

## 4 Discussion

As expected, COVID-19 was characterized by high levels of systemic inflammation, as reflected by the acute phase reactant, CRP, which is produced by the liver in response to increased expression of IL-6. Increased levels of CRP lead to elevated concentrations of IL-1β and TNF-α that, in turn, lead to the increased expression of adhesion molecules such as ICAM-1 (15). All the platelet and endothelial markers, with the exception of PDGF-BB, were significantly higher in people with COVID-19 than in the group of healthy controls, indicating the extent of platelet and endothelial activation elicited by SARS-CoV-2 (6).

Overall, levels of inflammation were equivalent between COVID-19 patients with and without HIV infection. While the levels of pro-inflammatory markers, such as CRP, IL-6, IL-1Ra, and TNF-α, were slightly higher, and anti-inflammatory cytokines, such as TGF-β1, were lower in COVID-19/PLWH than in those without HIV, none of these differences was significant. The platelet chemokine, RANTES, was significantly lower in PLWH but, after adjusting for other factors significantly associated with RANTES, such as age and sex, this difference lost significance. The initial difference observed was therefore likely due to the dissimilar demographic profiles of the patients with and without HIV infection.

The aforementioned systemic, pro-inflammatory biomarker data reflected, and probably explain, the essentially comparable clinical data, which characterized the groups of SARS-CoV-2-HIV-infected and –uninfected patients. While patients with COVID-19 without HIV infection appeared to have a more coordinated immune response, as reflected by the larger number of significant correlations between the immunological markers, this did not translate into differences in levels of inflammation or clinical outcomes. There were, however, a few exceptions. PLWH had lower levels of the acute phase reactant, ferritin, possibly secondary to less hypoxia (supported by the fact that PLWH needed less oxygen supplementation). In contrast, PLWH had higher levels of the coagulation marker, INR, together with non-significantly elevated levels of D-dimer. D-Dimer levels increase on recurrent cycles of coagulation and fibrinolysis, are associated with thrombosis and thromboembolic conditions and are, therefore, considered an important predictor of these events in COVID-19 patients (16). A correlation between D-Dimer and various physiological processes recognized in PLWH have been reported. These include endothelial dysfunction, microbial translocation, and active viral replication, as measured by VL (17–19). Interestingly, an equivalent number of clinical thrombotic events was reported in the two groups, although an important caveat is the absence of specialized scans that could have enabled diagnosis of occult thrombosis.

After multivariable logistic regression that accounted for age, gender, and differences in the prevalence of diabetes between the groups, VEGF emerged as a marker of interest. VEGF is a potent angiogenic factor and inducer of vascular permeability (20). It is released during hypoxia or in inflammatory conditions in response to endothelial injury and has been shown to be significantly increased in patients with COVID-19, correlating with disease severity (20, 21).

The early stage of endothelial activation is characterized by increased expression of ICAM-1 on the surface of endothelial cells to allow trans-endothelial migration of leukocytes to sites of inflammation (20). This is followed by elevation of plasminogen activator inhibitor-1 (PA-1) and soluble thrombomodulin that initiate alterations in the coagulation process, as well as VEGF, which drives angiogenesis (20). The interaction of these biomarkers with leukocytes, smooth muscle cells, and other proinflammatory cytokines, leads to remodeling of the vessel wall and subsequent vasculopathy (22). In this regard, it is interesting to note that VEGF was strongly and positively correlated with the pro-inflammatory cytokines, IL-1β, TNF-α and the chemokine, MIP-1α, in both groups, indicating the recruitment of inflammatory cells to the newly formed vessels.

HIV has long been known to be associated with higher levels of VEGF than uninfected controls (23). This is generally assumed to be detrimental and associated with the presence of Kaposi’s sarcoma, as well as HIV-associated encephalopathy (24). It has, however, also been postulated that VEGF may play a role in maintaining vasculature and protecting against the development of age-related cognitive decline, and may hence represent an appropriate response under conditions of hypoxia (25). In this regard, it is interesting to note that, in our study, VEGF was higher in PLWH but that their oxygen saturation was higher and FiO2 lower, indicating less hypoxia. This suggests that the association between VEGF and HIV is unrelated to hypoxia, a contention also supported by others (26). It is intriguing to consider whether the higher levels of VEGF observed in PLWH could be related to the lower oxygen requirement in this population.

It would seem that the elevated levels of VEGF were caused by the interaction between HIV and SARS-CoV-2, since levels were significantly higher in the presence of co-infection than with HIV infection alone. This interaction was, however, evident only in patients with good HIV control, since levels in the co-infected group with active viral replication had median VEGF levels at the lower limit of detection of the assay. This is further supported by the fact that the difference in VEGF between COVID-19/PLWH and control PLWH was only evident in the presence of an undetectable VL. It is therefore interesting to note that some authors have proposed that VEGF might be a biomarker of a more preserved immune system (27). In this regard, it is noteworthy that PLWH with a low CD4 count and unsuppressed VL, common features of those who had demised, had significantly lower levels of VEGF.

COVID-19/PLWH with a CD4 count ≤200 cells/μL could be distinguished from those with higher CD4 counts by lower levels of VEGF and higher levels of IL-6. While their inflammatory markers were similar, it is notable that all four of the COVID-19/PLWH who died were in this category. This finding should be considered in light of the significant lymphopenia observed in all COVID-19 patients in this study.

## 5 Conclusion

In conclusion, this study of patients hospitalized with COVID-19 demonstrated a relatively low mortality and good outcomes overall, both in people with and without HIV infection. Although significantly elevated, no significant differences were observed in the levels of cytokines, chemokines, and growth factors for platelet- and endothelial-associated markers between patients with and without HIV infection, which is in keeping with the clinical findings. The exception was VEGF, which was higher in PLWH. VEGF is a potent angiogenic factor, which could be advantageous under hypoxic conditions. Interestingly, VEGF concentrations were lower in PLWH with a low CD4 count and unsuppressed VL, which could be an indication that these patients are less able to mount an appropriate immune response.

## Data availability statement

The original contributions presented in the study are included in the article/**Supplementary Material**. Further inquiries can be directed to the corresponding author.

## Ethics statement

Ethics approval was obtained from the Research Ethics Committee of the Faculty of Health Sciences of the University of Pretoria (247/2020). The studies were conducted in accordance with the local legislation and institutional requirements. Written informed consent was obtained for participation from the participants or the participants’ legal guardians/next of kin.

## Author contributions

TR, FA and VU conceptualized the study. MM, ZB and HS prepared samples and performed the biomarker assays. FA, VU and ZB recruited participants and collected clinical data. MM, ZB, HS, RA and TR wrote the final manuscript. All authors contributed to the article and approved the submitted version.
