# Supplementary material for: Comparison of platelet-and endothelial-associated biomarkers of disease activity in people hospitalized with Covid-19 with and without HIV co-infection
Source: Front Immunol. 2023 Aug 14;14:1235914. doi: 10.3389/fimmu.2023.1235914 (PMC10461055; doi:10.3389/fimmu.2023.1235914)
Supplement: Supplementary file 1 [file DataSheet_1.docx]

Supplementary Material

COMPARISON OF PLATELET- AND ENDOTHELIAL-ASSOCIATED BIOMARKERS OF DISEASE ACTIVITY IN PEOPLE HOSPITALIZED WITH COVID-19 WITH AND WITHOUT HIV CO-INFECTION

**Mieke A. van der Mescht^1^, Helen C. Steel^1^, Zelda de Beer^1, 2^, Fareed Abdullah^3,4^, Veronica Ueckermann^3^, Ronald Anderson^1^, Theresa M. Rossouw^1, 2*^**

^1^ Department of Immunology, Faculty of Health Sciences, University of Pretoria, Pretoria, South Africa

^2^ Department of Family Medicine, Tshwane District Hospital, Pretoria, South Africa

^3^ Division for Infectious Diseases, Department of Internal Medicine, Steve Biko Academic Hospital and University of Pretoria, Pretoria, South Africa.

^4^ Office of AIDS and TB Research, South African Medical Research Council, Pretoria, South Africa.

*** Correspondence:**Theresa Rossouw
theresa.rossouw@up.ac.za

# Supplementary Figures and Tables

**Supplementary Table 1:** **COVID-19 severity classification used at Steve Biko Academic Hospital/ Tshwane District Hospital**

|  | Mild | Moderate | Severe |
| --- | --- | --- | --- |
| Saturation | > 94% on room air | 90-94% on room air or PaO 2 /FiO 2 ratio 200-300 | <90% on room air or PaO 2 /FiO 2 ratio <200 |
| Respiratory rate | < 25/min | >25/minute  < 30/minute | > 30/minute |
| Pulse (bpm) | < 120 | >120 |  |
| Temperature (°C) | 36-39 | >39 |  |
| Mental status | Normal | Normal | Confusion or altered mental state |
| Other |  |  | Hypotension or shock  Multi-organ failure |

**Supplementary Table 2: Parameters of respiratory function on admission**

| **Variable** | **COVID-19**  **PLWH**  **(n=37)** | **COVID-19 HIV-uninfected (n=137)** | **Reference ranges** | **P-value** |
| --- | --- | --- | --- | --- |
| Pulse (beats/min) | 101.5 (88 – 110) | 97 (86 – 106) | 60-100 | 0.2763 |
| Respiratory rate (breaths/min) | 22.5 (20 – 29) | 26 (20 – 30) | 12-16 | 0.4034 |
| Saturation on RA (%) | 94 (86 – 98) | 88 (82 – 96) | 94-98 | 0.0503 |
| pH | 7.46 (7.41 – 7.48) | 7.44 (7.41 – 7.5) | 7.35-7.45 | 0.9066 |
| FiO2 | 0.26 (0.21 – 0.44) | 0.525 (0.21 – 0.9) | 0.21 | **0.0165** |
| PF ratio | 128.8 (107.6 - 306.7) | 123.6 (76.9 - 219.1) | >300 | ​​0.1895 |

All variables are shown as median (interquartile range).

Abbreviations: room air (RA), fractional inspired oxygen (FiO2), PaO2/FiO2 (PF ratio).

Bold indicates significant values.

**Supplementary Figure 1: Correlation of CD4 count, HIV VL, and cytokines**

**Supplementary Table 3: Comparison of cytokine concentrations in pg/mL between patient and control groups by means of Kruskal Wallis and the post-hoc Dunn test**

| **Variable** | **COVID-19**  **PLWH**  **(Group 1)**  **(n=37)** | **COVID-19**  **HIV-uninfected**  **(Group2)**  **(n=137)** | **Control (Group 3) (n=9)** | **Group 1 vs 2** | **Group 1 vs 3** | **Group 2 vs 3** |
| --- | --- | --- | --- | --- | --- | --- |
| ICAM-1 | 153.88 (132.09 – 170.56) | 144.19 (121.61 – 183.81) | 112.23 (78.97-112.36) | 0.3500 | **0.0017** | **0.0016** |
| TGF-β1 | 7.35 (5.2 – 11.57) | 8.08 (5.82 – 11.85) | 5.02 (3.10-5.73) | 0.4572 | **0.0130** | **0.0069** |
| RANTES | 65.10 (51.62 – 104.93) | 98.93 (45.63 – 181.51) | 57.29 (36.51-60.35) | **0.0340** | 0.1730 | **0.0227** |
| IL-1β | 1.95 (1.46 – 2.66) | 1.95 (1.46 – 2.89) | 1.22 (0.98-1.71) | 0.4788 | **0.0114** | **0.0075** |
| IL-1Ra | 894.53 (725.59 – 1133.51) | 812.8 (631.27 – 1089.68) | 237.73 (214.08-407.8) | 0.1007 | **0.0001** | **0.0001** |
| IL-6 | 5.79 (1.82 – 12.0) | 4.32 (1.6 – 9.4) | 0.49 (0.3-0.54) | 0.2074 | **0.0001** | **0.0002** |
| IL-8 | 19.69 (15.32 – 28.36) | 17.5 (10.73 – 23.48) | 3.58 (2.8-6.47) | 0.0682 | **0.0001** | **0.0001** |
| MIP-1α | 4.10 (2.69 – 5.17) | 3.29 (2.47 – 4.72) | 1.3 (0.95-1.3) | 0.1499 | **0.0001** | **0.0001** |
| PDGF-BB | 460.42 (192.38 – 955.7) | 504.48 (253.69 – 1044.48) | 392.41 (31.74-494.58) | 0.2597 | **0.0480** | **0.0160** |
| TNF-α | 93.16 (73.41 – 118.40) | 83.87 (68.6 – 102.39) | 55.49 (50.67-68.6) | 0.0723 | **0.0001** | **0.0006** |
| VEGF | 32.16 (4.22 – 165.71) | 4.22 (4.22 – 87.07) | 4.22 (4.22-4.22) | 0.0723 | **0.0082** | **0.0354** |

All variables are shown as median (interquartile range).

Abbreviations: intracellular-adhesion molecule-1 (ICAM-1), transforming growth factor-β1 (TGF-β1), regulated-on activation, normal T-cell expressed and secreted (RANTES), interleukin (IL), IL1-receptor antagonist (IL-1Ra), macrophage inflammatory protein-1 alpha (MIP-1α), platelet-derived growth factor BB (PDGF-BB), tumor necrosis factor-α (TNF-α), vascular endothelial growth factor (VEGF).

Bold indicates significant values.
